# *SEL1L* SNP rs12435998, a predictor of glioblastoma survival and response to radio-chemotherapy

**DOI:** 10.18632/oncotarget.3611

**Published:** 2015-04-10

**Authors:** Marta Mellai, Monica Cattaneo, Alessandra Maria Storaci, Laura Annovazzi, Paola Cassoni, Antonio Melcarne, Pasquale De Blasio, Davide Schiffer, Ida Biunno

**Affiliations:** ^1^ Neuro-Bio-Oncology Center/Policlinico di Monza Foundation, Vercelli 13100, Italy; ^2^ Institute for Genetic and Biomedical Research, National Research Council, Milan 20138, Italy; ^3^ Department of Medical Sciences, University of Turin/Città della Salute e della Scienza, Turin 10126, Italy; ^4^ Department of Neurosurgery, CTO Hospital/Città della Salute e della Scienza, Turin 10126, Italy; ^5^ IRCCS-Multimedica, Milan 20138, Italy; ^6^ ISENET Stem Cell Bank, Milan 20138, Italy

**Keywords:** brain tumors, glioblastoma multiforme, SEL1L, genetic variant, prognosis

## Abstract

The *suppressor of Lin-12-like* (*C. elegans*) (*SEL1L*) is involved in the endoplasmic reticulum (ER)-associated degradation pathway, malignant transformation and stem cells.

In 412 formalin-fixed and paraffin-embedded brain tumors and 39 Glioblastoma multiforme (GBM) cell lines, we determined the frequency of five *SEL1L* single nucleotide genetic variants with regulatory and coding functions by a SNaPShot™ assay. We tested their possible association with brain tumor risk, prognosis and therapy.

We studied the *in vitro* cytotoxicity of valproic acid (VPA), temozolomide (TMZ), doxorubicin (DOX) and paclitaxel (PTX), alone or in combination, on 11 GBM cell lines, with respect to the SNP rs12435998 genotype.

The SNP rs12435998 was prevalent in anaplastic and malignant gliomas, and in meningiomas of all histologic grades, but unrelated to brain tumor risks. In GBM patients, the SNP rs12435998 was associated with prolonged overall survival (OS) and better response to TMZ-based radio-chemotherapy. GBM stem cells with this SNP showed lower levels of *SEL1L* expression and enhanced sensitivity to VPA.

## INTRODUCTION

Primary brain tumors consist of tumors with neuroepithelial and/or mesodermal origin. The former are prevailingly gliomas and represent the greatest part, whereas meningiomas are the most frequent mesodermal tumors. Gliomas can be subdivided in astrocytic and oligodendroglial and, for clinical purpose, in low grade and high grade tumors, among which Glioblastoma multiforme (GBM) is the most frequent tumor type. Gliomas are classified in four histologic grades in agreement with the World Health Organization (WHO) criteria [1]. The two main features of GBM are the phenotypic and genotypic heterogeneity, and the cell resistance to therapy.

GBM has a median overall survival (OS) of six months after surgery, 12.1 months after radiotherapy (RT) and 14.6 months after adjuvant chemotherapy (CHT) by temozolomide (TMZ) [2].

Gliomagenesis is a rather complicated process. It requires the existence of a proliferative neuroepithelial tissue, characterized by genotypic and phenotypic heterogeneity [1]. Mutation accumulation and epigenetic events are responsible for their final molecular asset.

The etiology of gliomas is largely unknown. Familial aggregation and identification of common and rare susceptibility genetic variants suggest the presence of genetic predisposition. The number of presently known variants accounts for only a small proportion of cases [3, 4] and, therefore, additional genetic variants need to be discovered by genome-wide association studies. However, the high genetic heterogeneity does not favor the identification of new options for a targeted therapy and a better prediction of patient survival time [5, 6].

The current glioma treatment is based on a combination of surgical resection and TMZ-based RT-CHT, although the highly invasive growth of these tumors is responsible for the lack of a local control [2, 7]. Conformational RT is only temporarily effective, since infiltrating and migrating cells escape its effect, whereas whole brain irradiation damages the normal nervous tissue [8]. The benefits of RT may not outweigh the side effects caused by radiation damage [9]. TMZ has proven to slightly improve the median OS by three months in GBM patients and it is more effective in patients with *MGMT* promoter hypermethylation [7]. The adjuvant treatment with procarbazine, lomustine (CCNU) and vincristine (PCV) combination gave some improvement on the progression free survival (PFS), but not on OS, in anaplastic astrocytomas and in pure and mixed oligodendrogliomas [10].

Meningiomas are benign tumors, mostly of WHO grade I; WHO grade II (atypical) and, especially, WHO grade III (anaplastic) tumors are much rarer and characterized by a less favorable prognosis.

The *suppressor of Lin-12-like* (*C. elegans*) (*SEL1L*) gene (OMIM 602329) is a putative tumor suppressor gene involved in the endoplasmic reticulum (ER)-associated degradation (ERAD) pathway implicated in tumor progression [11, 12]. Recently, *SEL1L* was found to be down-regulated in pancreatic ductal adenocarcinoma (PDA) [13, 14] by aberrant up-regulation of few microRNAs (hsa-mir-143, hsa-mir-155 and hsa-mir-223) that became potential therapeutic targets [15]. Interestingly, the single nucleotide polymorphism (SNP) rs12435998 in *SEL1L* intron 3 has been reported to modify the age at diagnosis of Caucasian nonsmoker PDA patients, for which it was proposed as a diagnostic and prognostic marker [14].

*SEL1L* is involved in several other neoplasia [16–20]. Its reduced protein expression by RNA interference increases GBM stem cell sensitivity to valproic acid (VPA) treatments [21]. *SEL1L* also plays a role in the neural stem cell self-renewal [22].

In the current study, we analyzed five genetic variants within *SEL1L* with potential regulatory and coding functions and their possible association with brain tumor risk, prognosis and response to therapies. Furthermore, we tested the influence of the SNP rs12435998 on the *in vitro* cytotoxicity of GBM cell lines treated with different pharmacological agents.

## RESULTS

### *SEL1L* genotyping in brain tumors

Five genetic variants were genotyped in 328 gliomas and 84 non-glial tumors from Caucasian patients with diagnosis of brain tumors. All frequencies were in Hardy-Weinberg equilibrium. Of the genetic variants analyzed, only the SNPs rs12435998 (c.–88T > C) and rs11499034 (p.Asp162Gly) were detected in both glial and non-glial tumor series.

In gliomas, the SNP rs12435998 was found with a minor allele frequency (MAF) of 100/644 (0.155) (Table 1). It was present in astrocytic, pure and mixed oligodendroglial tumors of all histologic grades, with higher frequency in anaplastic (20/104, 0.192) than in low grade tumors (16/138, 0.116) (*pF* = ns), and in GBMs (63/186, 0.168) (Tables 1–3). In the latter, no difference in the allelic frequencies was found between primary (61/360, 0.169) and secondary tumors (2/12, 0.167) (Table 3).

**Table 1:** Gene frequencies of *SEL1L* nucleotide genetic variants in gliomas

Sequence variation	Minor allele	PA (*N* = 30)*	DA (*N* = 26)*	AA (*N* = 14)*	GBM (*N* = 372)*	O (*N* = 90)*	AO (*N* = 74)*	OA (*N* = 18)*	AOA (*N* =16)*
c.–366T > C	C	0.00	0.00	0.00	0.01	0.00	0.014	0.00	0.00
c.–354T > C	C	0.00	0.00	0.00	0.01	0.00	0.014	0.00	0.00
c.341–88T > C	C	1(0.033)	4(0.167)	3(0.214)	63(0.169)	11(0.115)	12(0.162)	1(0.056)	5(0.31)
p.Asp162Gly	G	0.00	0.00	1(0.071)	6(0.0161)	5(0.056)	2(0.027)	0.00	0.00
p.Ser658Pro	C	0.00	0.00	0.00	0.00	0.00	0.00	0.00	0.00

**Abbreviations:** SEL1L, suppressor of Lin-12-like; PA, pilocytic astrocytoma; DA, diffuse astrocytoma; AA, anaplastic astrocytoma; GBM, glioblastoma multiforme; O, oligodendroglioma; AO, anaplastic oligodendroglioma; OA, oligoastrocytoma; AOA, anaplastic oligoastrocytoma.

* Number of alleles.

**Table 2:** Gene frequency of *SEL1L* SNPs rs12435998 and rs11499034 in glioma subtypes

Tumor subtype	Patients (*N*)*	SEL1L rs12435998	*p* value	Patients (*N*)*	SEL1L rs11499034	*p* value
Astrocytic tumors (WHO Grades I–IV)	444	71 (0.167)	Ns	444	7 (0.16)	Ns

Oligodendroglial tumors (WHO Grades II–III)	186	23 (0.123)	Ns	184	7 (0.38)	0.0072**

Oligoastrocytic tumors (WHO Grades II–III)	34	6 (0.176)	Ns	34	0 (0.00)	Ns

**Abbreviations:** SEL1L, suppressor of Lin-12-like; SNP, single nucleotide polymorphism; WHO, World Health Organization;

Ns, not significant.

* Number of alleles.

** Case-control study with control frequencies from Saltini *et al*, 2004 [24].

**Table 3:** Gene frequency of *SEL1L* SNPs rs12435998 and rs11499034 in gliomas according to WHO grading

Tumor type	Patients (*N*)*	SEL1L rs12435998 MAF	SEL1L rs11499034 MAF
**WHO Grade I**			
Pilocytic astrocytoma	15	1 (0.033)	0 (0.00)
**WHO Grade II**			
Diffuse and gemistocytic astrocytoma	12/13	4 (0.167)	0 (0.00)
Oligodendroglioma	48/49	11 (0.122)	5 (0.057)
Oligoastrocytoma	9/9	1 (0.056)	0 (0.00)
Total	69	16 (0.116)	5 (0.38)
**WHO Grade III**			
Astrocytoma	7	3 (0.214)	1 (0.71)
Oligodendroglioma	37	12 (0.162)	2 (0.27)
Oligoastrocytoma	8	5 (0.313)	0 (0.00)
Total	52	20 (0.192)	3 (0.029)
**WHO Grade IV**			
pGBM	180	61 (0.169)	6 (0.18)
sGBM	6	2 (0.167)	0 (0.00)
Total	186	63 (0.168)	6 (0.016)

**Abbreviations:** SEL1L, suppressor of Lin-12-like; SNP, single nucleotide polymorphism; WHO, World Health Organization; MAF, minor allele frequency; GBM, glioblastoma multiforme; pGBM, primary GBM; sGBM, secondary GBM.

* Number of alleles.

When compared to Italian healthy controls (MAF = 0.144) [23] or to European CEU individuals (MAF = 0.181, NCBI), the allelic frequency observed in gliomas was not distributed in a significantly different way. The SNP rs12435998 was always found in heterozygosity with the exception of one pGBM (CTO3), carrier of the *CC* genotype, and originating in culture both NS and AC. In non-glial tumors, the SNP rs12435998 was identified in all represented tumor types, with higher frequencies in ependymomas (7/20, 0.35) and meningiomas (21/102, 0.206) (Table 4).

**Table 4:** Gene frequencies of *SEL1L* nucleotide genetic variants in non-glial tumors

Nucleotide variation	Minor allele	Schwannoma (*N* = 30)*	Ependymoma (*N* = 20)*	Meningioma (*N* = 102)*	Medulloblastoma (*N* = 16)*
c.–366T > C	C	0.00	0.00	0.00	1(0.063)
c.–354T > C	C	0.00	0.00	0.00	1(0.063)
c.341–88T > C	C	5(0.167)	7(0.350)	21(0.206)	1(0.063)
p.Asp162Gly	G	2(0.067)	0.00	0.00	1(0.063)
p.Ser658Pro	C	0.00	0.00	0.00	0.00

**Abbreviations:** SEL1L, suppressor of Lin-12-like.

* Number of alleles.

The SNP rs11499034 was detected in glial tumors with MAF of 14/640 (0.0218) (Table 1). It was found in pure oligodendroglial tumors (7/184, 0.038) of both histologic grades, as well as in malignant astrocytic tumors (7/386, 0.0181) (Tables 1–3). Interestingly, one pGBM (CV20) generating NS was found to be compound heterozygous for the SNPs rs12435998 and rs11499034. When compared with Italian healthy controls (MAF = 0.010) [24] or to European CEU individuals (MAF = 0.013, NCBI), the SNP rs11499034 allelic frequency in oligodendroglial tumors was not differently distributed (*pF* = ns). In non-glial tumors, the SNP rs11499034 was identified in Schwannomas (2/30, 0.067) and medulloblastomas (1/16, 0.063) (Table 4). It was always found in heterozygosity.

The two c.–366T > C and c.–354T > C genetic variants in the promoter region were rare variants (MAF < 0.01), in complete linkage disequilibrium. They were identified in one anaplastic oligodendroglial tumor and in one pGBM among gliomas and in one medulloblastoma within non-glial tumors (Tables 2, 4).

The p.Ser658Pro missense variation was never found in both tumor series.

The constitutional nature of each polymorphic SNP was demonstrated by comparison of matched tumor and blood/saliva samples from 41 patients.

### Relationship of the SNP rs12435998 with clinical and molecular features

The SNP rs12435998 was not associated with sex, patient age (≤ 50 or > 50 years), tumor location or age at diagnosis both in glial and non-glial tumors (*p* > 0.05 for all categories).

In low and high grade gliomas of the present series, the frequency of the SNP rs12435998 was compared with a series of genetic and epigenetic alterations *(IDH1/2* somatic mutations, *EGFR* gene amplification, *MGMT* promoter hypermethylation, *TP53* mutations or *TERT* promoter mutations [personal data], 1p/19q chromosome status, already published [25–28]. No association was found with any of them (*p* > 0.05 for all).

### SEL1L genotyping in GBM cell lines

All genetic variants were genotyped in the panel of 39 GBM cell lines and in two neuralized pluripotent human control cell lines.

The SNPs rs12435998, rs11499034 and the c.–366T > C and c.–354T > C genetic variants found in tumors were also detected in the derived GBM cell lines. Out of the 16 NS, seven (CV1 NS, CV10 NS, CV20 NS, NO4 NS, CTO3 NS, CTO15 NS and 010627 NS) carried the SNP rs12435998, one (CV20 NS) also the SNP rs11499034 whereas another one (CTO5 NS) the two c.–366T > C and c.–354T > C genetic variants (supplementary Table S1). Six out of the former seven (CV1 NS, CV10 NS, CV20 NS, NO4 NS and CTO15 NS and 010627 NS) carried the rs12435998 *TC* genotype whereas the other (CTO3 NS) the *CC* genotype. The rs11499034, c.–366T > C and c.–354T > C variants were in a heterozygous status. Out of the 18 AC, eight (CV4 AC, CV6 AC, CV9 AC, CV10 AC, NO2 AC, NO4 AC, CTO15 AC and 010627 AC) carried the rs12435998 *TC* genotype and one (CTO5 AC) both the c.–366T > C and c.–354T > C variants in a heterozygous status. Within the five cell lines from coated matrix, one (G179) displayed the rs12435998 *TC* genotype whereas the two neuralized pluripotent human control stem cell lines carried the wild type genotype (supplementary Table S1).

### *In silico* analysis of *SEL1L* genetic variants

By *in silico* analysis, the p.Asp162Gly (rs11499034) missense variation was predicted to be possibly damaging by PolyPhen-2, contrarily to SNAP and pMUT tools predicting a neutral effect.

For the SNP rs12435998, two distinct bioinformatic tools (ESE Finder and Human Splicing Finder) predicted a possible functional effect since the minor allele *C* creates a putative exonic splicing enhancer (ESE) motif responsive to the serine/arginine-rich (SR) protein SC35. The nucleotide variant generates a *cis* sequence element important for both the correct splice site identification and the regulation of alternative splicing. This ESE motif acts as binding site for the splicing factor SC35, a member of the spliceosome required for the formation of the earliest ATP-dependent splicing complex.

### Survival analysis

**Figure 1:**
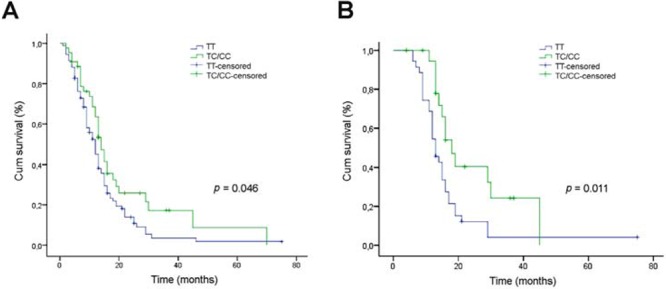
Relationship between the *SEL1L* SNP rs12435998 and survival in GBM patients **A.** Kaplan-Meier survival curves for overall survival (OS) in GBM patients with respect to the *SEL1L* SNP rs12435998 genotype. **B.** Kaplan-Meier survival curves for OS in the subgroup of 55 GBM patients treated with TMZ-based RT-CHT with respect to the SEL1L SNP rs12435998 genotype. **Abbreviations:** SEL1L, suppressor of lin-12-like; SNP, single nucleotide polymoprphism; GBM, glioblastoma multiforme; OS, overall survival; SNP, single nucleotide polymorphism; Cum, cumulative.

The relationship of the SNP rs12435998 with OS was evaluated in 137 GBM patients and in 58 patients with oligodendroglial neoplasia. In the former, the SNP rs12435998 was identified in 44 patients (48/274, 0.175), including 40 *TC* heterozygote and 4 *CC* homozygote cases that were considered as a single group. By univariate analysis, the SNP rs12435998 minor allele *C* improved patient OS (*p* = 0.046, Log Rank test), regardless of the post-surgical treatment (Figure 1A). The median survival time was 14 months for *TC*/*CC* genotype carriers and 12 months for wild type patients. Censored cases were 12/44 (0.273) for the former and 12/93 (0.129) for the latter. In the group of 55 patients treated according to Stupp's protocol [2], the SNP rs12435998 minor allele *C* was significantly associated to a better response to TMZ based RT-CHT (*p* = 0.011, Log Rank test) (Figure 1B). The median survival time was 18 months for *TC*/*CC* genotype carriers and 13 months for wild type patients. Censored cases were 7/20 (0.35) for the former and 3/35 (0.086) for the latter. With respect to the *MGMT* promoter hypermethylation status in the same group of patients, the SNP rs12435998 minor allele *C* improved OS in both methylated and unmethylated cases, more evidently in the former than in the latter (data not shown).

The SNP rs12435998 minor allele *C* did not improve patient OS (*p* = 0.540, Log Rank test) in the subgroup of 34 patients that received RT as single treatment (data not shown).

In oligodendroglioma patients, the SNP rs12435998 minor allele *C* was identified in 17 patients (34/116, 0.293), including 16 *TC* heterozygote and 1 *CC* homozygote cases that were considered as a single group. Univariate analysis revealed that the SNP rs1243599 did not affect patient OS (data not shown). Censored cases for wild type patients were 8/17 (0.47) and 26/41 (0.63) for *TC*/*CC* genotype carriers, respectively.

### *SEL1L*, *SOX2*, *NOTCH1* and *Gadd45β* expression in GBM cell lines with respect to the SNP rs12435998 and culture conditions

**Figure 2:**
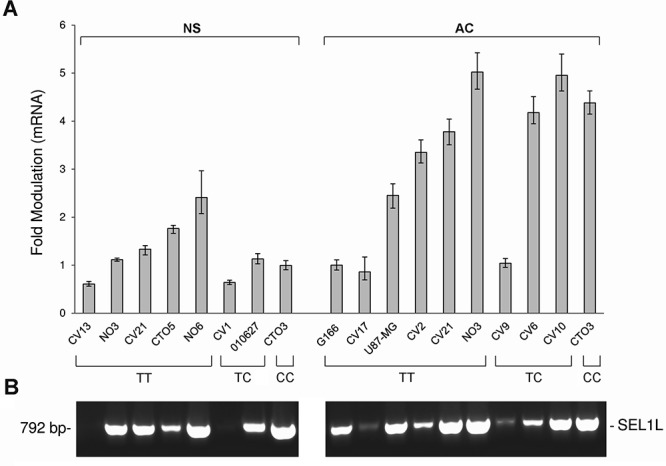
SEL1L expression levels by qRT-PCR and analysis of *SEL1L* alternative transcripts in GBM cell lines **A.** *SEL1L* expression levels in NS and AC cultures with respect to the *SEL1L* SNP rs12435998 genotype. **B.** Analysis of *SEL1L* alternative transcripts in the same panel of GBM cell lines with respect to the *SEL1L* SNP rs12435998 genotype. **Abbreviations:** SEL1L, suppressor of lin-12-like; qRT-PCR, quantitative real time (qRT)-PCR; GBM, glioblastoma multiforme; SNP, single nucleotide polymorphism; NS, neurospheres; AC, adherent cells.

**Figure 3:**
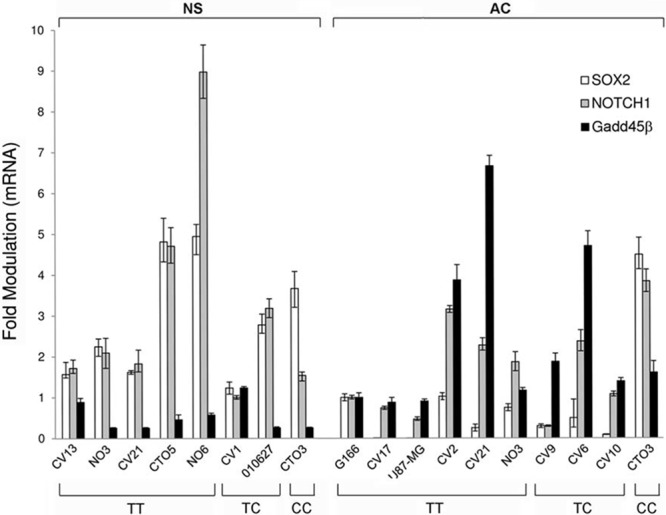
*SOX2*, *NOTCH1* and *Gadd45β* expression levels by qRT-PCR in GBM cell lines *SOX2*, *NOTCH1* and *Gadd45β* expression levels by qRT-PCR in the same panel of GBM cell lines (NS and AC) with respect to the *SEL1L* SNP rs12435998 genotype. **Abbreviations:** qRT-PCR, quantitative real time (qRT)-PCR; GBM, glioblastoma multiforme; SNP, single nucleotide polymorphism; NS, neurospheres; AC, adherent cells.

*SEL1L* expression was evaluated in 18 GBM cell lines carrying different genotypes of the SNP rs12435998, in order to evaluate the influence on the mRNA. Five of these (CV1 NS, CV13 NS, CTO5 NS, NO6 NS and 010627 NS) were derived and grew as NS, six (CV2 AC, CV6 AC, CV9 AC, CV10 AC, CV17 AC and U87-MG AC) as AC, three (CV21, CTO3 and NO3) both as AC and NS, and one (G166) in coated matrix. Lower *SEL1L* mRNA expression levels were observed in NS compared to AC, and the minor allele *C* appeared to be associated in the former to lower *SEL1L* expression levels (Figure 2A). We also evaluated *SOX2* and *NOTCH1* gene expression and we found higher levels in NS compared to AC (Figure 3), with the exception of CTO3 cell line (carrying the *CC* genotype) where their expression was always high in both NS and AC. In contrast, the *Gadd45β* gene expression was higher in AC than in NS (Figure 3).

*SEL1L* did not generate a spliced transcript as demonstrated by the absence of additive amplified PCR products using a primer set designed against *SEL1L* cDNA flanking the intron 3 (Figure 2B).

### SEL1L protein expression with respect to the SNP rs12435998

**Figure 4:**
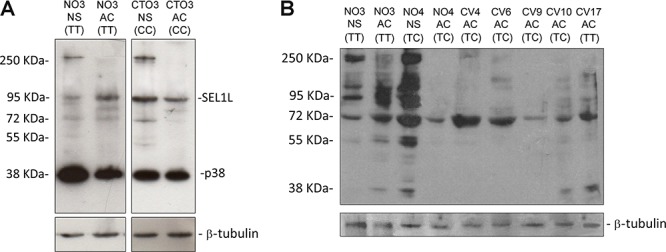
Western blotting analysis for SEL1L in GBM cell lines *SEL1L* expression in NS and AC with respect to the *SEL1L* SNP rs12435998 genotype using a monoclonal **A.** and a polyclonal **B.** anti-SEL1L antibody. **Abbreviations:** SEL1L, suppressor of lin-12-like; NS, neurospheres; AC, adherent cells; SNP, single nucleotide polymorphism; GBM, glioblastoma multiforme; NS, neurospheres; AC, adherent cells.

Western blotting analysis performed on 11 GBM cell lines with a mouse monoclonal and a goat polyclonal anti-SEL1L antibody raised against the N-terminus region revealed the presence of additive bands in NS, carrying either the rs12435998 wild type or *CC* genotype (Figure 4A, 4B). Indeed, besides the well-known ER anchored protein with molecular weight of 95 kDa and the smaller secreted variant of 38 kDa, an additional band of approximately 260 kDa is clearly visible in NS but not in AC, regardless of the nucleotide variant, using both the monoclonal and the polyclonal anti-SEL1L antibody (Figure 4A, 4B).

### Drug treatments and cytotoxicity assay

**Figure 5:**
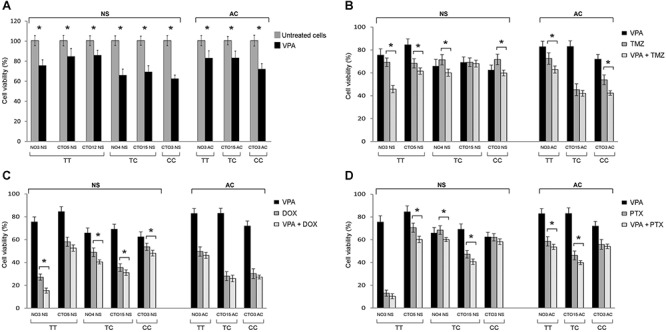
Effect of the SEL1L SNP rs12435998 on the response to drug treatments in GBM cell lines **A.** Inhibitory effect of 2 mM VPA alone on the proliferation of NS and AC treated for 96 h. Effect of combination of 2 mM VPA with 50 μM TMZ **B.** with 1 μM DOX **C.** and with 50 nM PTX **D.** after 96 h exposure. Significant enhanced anti-proliferative effect is indicated with*. Data are average values ± standard deviation (SD) of three independent experiments, each performed in triplicate. **Abbreviations:** SEL1L, suppressor of Lin-12-like; SNP, single nucleotide polymorphism; GBM, glioblastoma multiforme; VPA, valproic acid; TMZ, temozolomide; DOX, doxorubicin; NS, neurospheres; AC, adherent cells; SNP, single nucleotide polymorphism; NS, neurospheres; AC, adherent cells.

By MTT analyses and cell counts VPA displayed a dose-dependent inhibitory effect on the *in vitro* proliferation of all GBM cell lines, both NS and AC (unpublished data). A drug concentration of 2 mM allowed to significantly reduce the viability of all tested cell lines. Moreover, the cytotoxic action of the drug appeared more evident on NS that on the correspondent AC from the same tumor (where it was available). We observed that NS carrying either the *TC* or the *CC* genotype showed a higher sensitivity to VPA (32.55% and 37.6% viability reduction, respectively) compared to NS with wild type genotype (18.14% average viability reduction from four different cell lines). In AC, a relationship between VPA cell response and genotype was not so evident, even if the sample size does not allow meaningful conclusions (Figure 5A).

The combined treatment of VPA with TMZ, DOX or PTX resulted in a significant increase of the anti-proliferative effect compared to single treatments on the most cell lines. The range of viability reduction for the combined treatment in comparison with the drug alone was 1.72%–33.94% (min value-max value) for VPA + TMZ, 6.78%–27.91% for VPA + DOX, 3.06–14.63% for VPA + PTX, regardless of the culture conditions (Figure 5B–5D). Compared to the control, the average viability reduction for the combined treatment with respect to NS and AC culture conditions was 15.60% and 13.90%, respectively, for VPA + TMZ, 15.46% and 7.93% for VPA + DOX and 10.86% and 8.38% for VPA + PTX.

The greatest anti-proliferative effect was found for the combined treatment of VPA + TMZ. It was significantly more effective than TMZ alone in all cell lines, with the exception of one case (CTO15 NS and CTO15 AC). The enhancement of the response was especially observed in NS with unmethylated *MGMT* and wild type *TP53* genes. No association has been found, however, between the SNP rs12435998 genotype and either the *MGMT* hypermethylation status or the *TP53* mutation status (supplementary Table S1).

The association of VPA with DOX and PTX resulted in an increased anti-tumor effect, even if it was not verified in all cell lines.

The occurrence of the SNP rs1435998 influenced, in NS, the response to VPA alone whereas it did not affect the response to TMZ, DOX and PTX as either single or combined treatment. In AC, the SNP rs1435998 appeared to enhance the response to TMZ and DOX, alone or in combination with VPA, although this observation is limited to one cell line representative of each genotype (Figure 5B, 5C).

## DISCUSSION

Our investigation started from the assumption that *SEL1L* is a putative tumor suppressor gene involved in the ERAD pathway and in the neoplastic progression [11, 12]. *SEL1L* has recently been reported to be significantly down-modulated in human PDA by aberrant microRNA up-regulation [13–15]. In gliomas, *SEL1L* is down-regulated as well, even though its mechanism is still unclear. It must be recalled that *SEL1L* SNP rs12435998 was previously demonstrated to be an independent susceptibility factor in Alzheimer's disease [23].

In this study we genotyped five genetic variants within *SEL1L* selected for their potential regulatory and coding functions in a series of 412 human brain tumors and 39 GBM cell lines.

We detected the two SNPs rs12435998 and rs11499034 in both glial and non-glial tumor series. The SNP rs12435998 was detected in astrocytic and oligodendroglial gliomas, with a higher frequency in malignant than in benign tumors. Its allelic frequency, however, was not significantly different either in comparison to controls or comparing benign to malignant tumors.

In non-glial tumors, the SNP rs12435998 was detected in all tumor types, but it prevailed in meningiomas and ependymomas, without a statistically significant difference when compared to controls. This finding is in line with the significant association between an intergenic region 600 kb 5′ to *SEL1L* and meningioma progression recently described with two independent SNP cohorts [29]. In our series, however, the occurrence of the SNP rs12435998 did not correlate with the tumor grade. As a matter of fact, *SEL1L* maps on the critical region 14q31.3, which typically displays LOH in atypical and, more frequently, in anaplastic meningiomas [30]. Its prevalence in meningiomas might also be interpreted in relation to their possible origin from the neural crest, as suggested at variance with the mesodermal origin [31].

In GBM patients, the survival analysis revealed a significantly longer median survival time in *TC/CC* variant genotype carriers compared to wild type patients, regardless of the post-surgical treatment and more evidently, after TMZ-based RT-CHT, both in *MGMT* methylated and unmethylated cases. The influence of the SNP rs12435998 did not result in the subgroup of 34 patients that were treated with RT alone since diagnosed before the Stupp's protocol approval. These observations thus legitimize the discussion of the SNP rs12435998 as a novel prognostic and predictive marker for GBM patients.

In the oligodendroglioma group, most patients are still alive and no statistically significant result was obtained from survival analysis. As it is known, the effect of therapies on gliomas depends on the balance between sensitivity and resistance. The former is linked to the DNA exposure during replication, so that malignant gliomas are more sensitive to DNA damages by RT or TMZ. This explains why therapies are efficient on malignant gliomas, even though they display several resistance mechanisms, and why, within patients affected by oligodendrogliomas, which are less resistant and, at the same time, less malignant, the number of censored cases is high. It would be interesting to explore the frequency of this SNP in tumors characterized by a more prolonged OS and better prognosis (such as breast, colorectal, lung, esophageal and prostatic cancers) in order to highlight an association more pronounced with the tumor outcomes.

In spite of the possible functional effect of the SNP rs12435998 deduced by the *in silico* prediction, the analysis of *SEL1L* alternative transcripts on GBM cell lines carrying either the *TC* or *CC* variant genotype did not reveal aberrant splicing. Notably, the putative ESE motif predicted by two distinct bioinformatic tools (ESEfinder and Human Splicing Finder) acts as binding site for the splicing factor SC35, a member of the spliceosome required for the formation of the earliest ATP-dependent splicing complex and known to enhance the splicing of the Protein Kinase C δVIII (PKCδVIII) isoform in neurons [32], to play a potential role in neurogenesis, chromatin remodelling and self-renewal capacity of embryonic stem cells [33].

The SNP rs1149934 was detected in anaplastic and malignant astrocytic tumors, as well as in pure oligodendrogliomas, without association with brain tumor risk. Interestingly, the pGBM cell line heterozygous for this SNP (CV 20 NS), only developed in NS condition, suggesting a potential functional effect of this nucleotide variant on cell-cell and cell-matrix adhesion likely *via* fibronectin. The two single nucleotide variants c.–366T > C and c.–354T >C in the promoter region are rare variants, identified one for each tumor series. The missense variation p.Ser658Pro in exon 19 was never found in both series.

In GBM cell lines considered as either glioma initiating stem cells (GISCs) or glioma-associated stem cells (GASCs) [34], the assessment of the expression levels of *SEL1L* mRNA revealed higher level of transcript in AC compared to NS, regardless of the SNP rs12435998 genotype. This is in agreement with the differentiation of the former and the dedifferentiation of the latter, as previously demonstrated in murine neural stem cell lineage commitment [22] and in tumor transformation [16–20]. This finding is also consistent with the higher *SOX2* and *NOTCH1* expression, both markers of stemness, observed in NS compared to AC. An exception is represented by CTO3 NS and CTO3 AC, carriers of the homozygous *CC* variant genotype, both expressing marked levels of the two genes. In fact, they share the same molecular profile of the respective primary tumor. This was interpreted as due to the occurrence in AC of a spectrum of maturation shifted towards stemness. The higher expression of *Gadd45β* found in general in AC compared to NS, correlates with their reduced proliferative potential.

Moreover, NS displayed by Western blotting analysis a supplementary band of 260 kDa that was not novel in our experience (IB, personal observations). It may represent a *SEL1L* precursor possibly related to the stemness property of NS, since it is not expressed in AC.

Within GBM cell lines, the SNP rs12435998 down-regulates *SEL1L* expression only in NS and, at the same time, it increases the sensitivity to VPA, confirming a previous observation [21]. In fact, compared to wild type NS, the cell viability after VPA was significantly lower in NS with the *TC/CC* variant genotype. It is well known that nucleosome dense genome regions play a crucial role in controlling the elongation rate of transcription machinery and in regulating access of the promoter to transcription factors [35, 36]. Based on the large extension of intron 3 (20491 nt), we speculate that the minor allele *C* alters the nucleosome genome landscape with subsequent destabilization of exon-intron junctions contributing to remarkable variation of the state–state mRNA levels. Moreover, the potential change in nucleosome packing might create a more permissive or restrictive state for the binding of specific *SEL1L* repressor or activator related to stenmness such as previously described SC35.

In GBM cell lines, combined therapies of VPA with TMZ, DOX and PTX are more effective than the drugs alone. VPA appear able to potentiate the cytotoxicity and to improve the efficacy of the other chemotherapeutics, especially of TMZ, and more in NS than in AC. In general, the effect of a combined therapy on GBM cell lines depends on the action mechanisms of the various drugs, in association with the genetic and epigenetic asset of each cell line. As for TMZ, this effect should be interpreted according to both the differential hypermethylation status of the *MGMT* promoter region and the *TP53* mutation status of the cell lines [37].

Our findings are consistent with the demonstrations that VPA is effective on GBM cell lines through inhibition of histone deacetylases (HDACs) [38] and that combined VPA + TMZ treatment is effective on glioma cell lines, mainly on TMZ-resistant ones, where VPA down-regulates at protein level the expression of the MGMT [39, 40].

The combined treatment of VPA with conventional TMZ-based RT-CHT has been previously proved to prolong survival in GBM patients [40], in line with the less recent demonstration that combined therapy could also be useful in malignant pediatric brain tumors [41]. In GBM patients, the combination of VPA with RT and CHT may thus appear as a rationale therapeutic option [42].

The potential relationship between the SNP rs12435998 and the increased response to TMZ found in AC may be in line with its predictive effect on our series of GBM patients.

In conclusion, this is the first study reporting a significant association of the *SEL1L* SNP rs12435998 constitutive genetic variant with an improved OS in GBM patients, especially after conventional treatment with TMZ-based RT-CHT.

*In vitro,* we also demonstrated that the SNP rs12435998 down-regulates *SEL1L* expression in NS cultures derived from pGBMs and that it sensitizes these cells to VPA treatment.

The main limitation of the research is the low number of GBM cell lines used to test the *in vitro* cytotoxicity. Further studies are needed to confirm the putative association of the SNP rs12345998 with the response to standard TMZ-based RT-CHT on independent series of GBM patients and the *in vitro* response to VPA and TMZ, alone or in combination, in a larger panel of GBM cell lines.

Future implications of our observations include the possibility to test the SNP rs12435998 in the diagnostic practice of malignant gliomas and to consider the combined use of VPA and TMZ in the post-surgical therapeutic strategies of GBM patients.

## MATERIALS AND METHODS

### Ethics statement

Primary human GBM specimens for molecular genetics and cultures were obtained and used in compliance with the local institutional review board and Committee on Human Research and with the ethical human subject principles of the World Medical Association Declaration of Helsinki Research. Written informed consent was obtained from all patients after appropriate ethics approval of the CTO Hospital/Città della Salute e della Scienza (n. 487/2012).

### Brain tumor specimens

A total of 412 brain tumors (328 gliomas and 84 non-glial tumors) were analyzed. Three hundred eighty-one formalin-fixed paraffin-embedded (FFPE) gliomas were obtained from the archive material of the Department of Medical Sciences of University of Turin/Città della Salute e della Scienza (Turin, Italy). The eighty-four FFPE non-glial tumors were obtained from the archive material of the Neuro-Bio-Oncology Center/Policlinico di Monza Foundation (Vercelli, Italy) (Table 5). Patients underwent either partial or total resection. The histologic diagnosis was performed according to the WHO guidelines [1]. GBMs were classified as primary (pGBM) or secondary tumors (sGBM) according to a previous histologically verified low grade glioma. From 41 patients, a matched constitutional DNA from blood/saliva was available as well.

**Table 5:** Patient demographics

Tumor type	WHO grading	Patients (*N* )	Gender (M/F)	Mean age (years) and range
**Glial tumors (*N* = 328)**				
**Pilocytic astrocytoma**	I	15	8/7	33 (9–68)
**Diffuse and gemistocytic astrocytoma**	II	13	6/7	42 (23–68)
**Anaplastic astrocytoma**	III	7	7/0	49 (24–75)
**Primary GBM**	IV	180	112/68	60 (23–83)
**Secondary GBM**	IV	6	4/2	47 (42–52)
**Oligoastrocytoma**	II	9	7/2	41 (31–53)
**Anaplastic oligoastrocytoma**	III	8	4/4	52 (37–71)
**Oligodendroglioma**	II	49	28/21	47 (26–79)
**Anaplastic oligodendroglioma**	III	41	22/16	55 (31–80)
**Non-glial tumors (*N* = 84)**				
**Meningioma**	I	38	19/19	63 (48–78)
**Meningioma**	II	8	5/3	58 (36–79)
**Meningioma**	III	5	2/3	64 (54–80)
**Schwannoma**	I	15	9/6	59 (25–82)
**Ependymoma**	III	10	8/2	63 (48–84)
**Medulloblastoma**	IV	8	6/2	26 (8–39)

**Abbreviations:** WHO, World Health Organization.

Supplementary thirty-one brain tumors (22 gliomas and 9 non-glial tumors) were made available to us by Dr. Marc Sanson from Pitie-Salpétrière Hospital (Unite INSERM U495, 2. Federation de Neurologie Mazarin, Paris, France) (Table 5). They were diagnosed as above mentioned and selected based on the evidence of loss of heterozygosity (LOH) on chromosome 14q31.3 [43], *SEL1L* location site. Patient demographics are summarized in Table 5.

### Cell lines

A total of 30 cell lines derived from surgically resected pGBMs were studied. Culture conditions have previously been described [25, 44].

Fourteen cell lines (CV1 NS, CV7 NS, CV10 NS, CV13 NS, CV17 NS, CV20 NS, CV21 NS, NO3 NS, NO4 NS, NO6 NS, CTO3 NS, CTO5 NS, CTO12 NS and CTO15 NS) were isolated in Dulbecco's modified Eagle's medium (DMEM)/F-12 supplemented with 20 ng/mL epidermal growth factor (EGF) and 10 ng/mL basic fibroblast growth factor (bFGF) for neurosphere (NS) assay (NSA). Sixteen cell lines (CV2 AC, CV3 AC, CV4 AC, CV6 AC, CV8 AC, CV9 AC, CV10 AC, CV17 AC, CV21 AC, NO2 AC, NO3 AC, NO4 AC, CTO3 AC, CTO5 AC, CTO12 AC and CTO15 AC) developed in DMEM with 10% fetal bovine serum (FBS) for conventional monolayer growth and named adherent cell (AC). Both cultures were maintained in a 5% O_2_ and 5% CO_2_ humidified atmosphere.

Cell line authentication from the respective primary tumor was obtained by Short Tandem Repeat (STR) profiling.

Two malignant glioma cell lines (U87-MG and 010627) were kindly supplied by Dr. Rossella Galli (DIBIT San Raffaele, Milan, Italy) and maintained as both NS and AC.

Furthermore, five GBM cell lines (GliNS2, GBM2, G144, G166, G179) and two neuralized pluripotent human control stem cell lines (CB660 [fetal] and H9 [embryonic]), kindly supplied by ISENET Stem Cell Biobank (www.isenet.it), were cultured in coated matrix according to Pollard's, Sun's and Cattaneo's conditions, respectively [21, 45, 46].

All experiments on GBM cell lines were carried out with cells from passages 10–20. All cultures were checked for *Mycoplasma* contamination before experimental use (e-Myco™ Mycoplasma PCR Detection kit, iNtRON Biotechnology, Korea).

### Glioma patient stratification

Survival data were available for 137 GBM patients. Out of these, 89 patients received post-operative conventional fractionated RT (60 Gy total dose; 2 Gy × 5 days/week for 6 weeks). Fifty-five of these received concomitant CHT with TMZ (75 mg/m^2^/daily for 6 weeks) followed by adjuvant TMZ (200 mg/m^2^ × 5 days/week every 4 weeks for 6–12 cycles) according to the EORT/NCIC-regime published by Stupp *et al* [2]. Thirty-four patients received RT and four CHT as single treatment with the above mentioned schedule. Fourteen patients had no post-surgical treatment whereas 30 cases were lost at follow-up.

Survival data were available for 58 oligodendroglioma patients (33 WHO grade II and 25 WHO grade III tumors). Out of these, sixteen received postoperative conventional fractionated RT (60 Gy total dose; 2 Gy × 5 days/week for 6 weeks). Fourteen of them also received either concomitant CHT with TMZ (75 mg/m^2^/daily for 6 weeks) or adjuvant TMZ (200 mg/m^2^ × 5 days/week every 4 weeks for 6–12 cycles) or both. Eight patients received TMZ (200 mg/m^2^ × 5 days/week every 4 weeks for 6–12 cycles) only, whereas five patients had no post-surgical treatment. Twenty-nine patients were lost at follow-up.

### DNA extraction

Genomic DNA (gDNA) from FFPE tumor samples and cell lines was isolated using the QIAamp DNA Mini kit (Qiagen NV, Venlo, The Netherlands). Constitutional gDNA from peripheral blood was extracted by a salting-out protocol whereas from saliva with the Oragene DNA Collection kit (DNA Genotek Inc., Ontario, Canada, USA).

As reference control group, a series of 153 (59 men and 94 women, mean age 66.8 ± 11.93 years) unrelated, healthy and ethnicity-matched individuals was collected from medical students, university/hospital staff and blood donors [23, 24]. gDNA from this series was extracted using a silica-based method [48].

### Single nucleotide genetic variant selection

The *SEL1L* gene spans more than 62.24 kb pairs within a “gene desert region” and displays only weak linkage disequilibrium pattern according to the HapMap CEU population data. A total of five single nucleotide genetic variants in the *SEL1L* gene (GenBank Reference sequence NM_005065) were selected (Table 6). Two of them (c.–366T > C and c.–354T > C) were first identified in the minimal promoter region of the *SEL1L* gene in lung carcinoma patients [47]. The c.341–88T > C genetic variant is a common SNP (rs12435998) within intron 3, containing potential binding sites for transcription factors involved in ER-induced stress, and it is a predicted splice site [23]. The c.485A > G (p.Asp162Gly) variant corresponds to the SNP rs11499034, maps in exon 4 encoding for the fibronectin type II domain (FN2), and affects a highly conserved amino acid residue [24]. The amino acid change from Asp to Gly may have a disruptive role in the collagen binding. The c.1972T > C (p.Ser658Pro) variant in exon 19 has been described as somatic mutation responsible for the progressive early-onset cerebellar ataxia in canine species [49]. Of note, the *SEL1L* gene is highly conserved between dog and human, with 98% identity at protein level (HomoloGene). The Ser658 residue located in the eleventh *SEL1L*-like repeat is completely conserved in all aligned vertebrates and maps within a functionally relevant domain for tumor growth inhibition [50].

**Table 6:** *SEL1L* nucleotide genetic variants analyzed in the current study

Nucleotide change	Amino acid change	Location	SNP ID	Reference	PCR primers from 5′ to 3′ (forward/reverse)
**c.–366T > C**	-	Promoter	-	[47]	CTTGTGAATCCATAGCCTTGA/ATTGTACGAAGCTCCCACAC
**c.–354T > C**	-	Promoter	-	[47]	CTTGTGAATCCATAGCCTTGA/ATTGTACGAAGCTCCCACAC
**c.341–88T > C**	-	Intron 3	rs12435998	[23]	TGCTAGCCCTTTTATGTTCC/TGCCCTAGACATAAAGCAATG
**c.485A > G**	p.Asp162Gly	Exon 4	rs11499034	[24]	TGCTAGCCCTTTTATGTTCC/TGCCCTAGACATAAAGCAATG
**c.1972T > C**	p.Ser658Pro	Exon 19	-	[48]	AGCATGTCAATGGGAGGAG/TGCAAGTATTTTCCCCAATC

**Abbreviations:** SEL1L, suppressor of Lin-12-like; WHO, World Health Organization; SNP, single nucleotide polymorphism; PCR, polymerase chain reaction.

### Genotyping

Single nucleotide variant genotyping was performed by a SNaPShot™ SNP Multiplex Genotyping assay (Thermo Fisher Scientific Inc., Waltham, MA, USA) according to the manufacturer's instructions. Genetic regions flanking each genetic variant were co-amplified using two multiplex polymerase chain reaction (PCR) primer pools (Table 6). PCR amplification was performed in a total volume of 12.5 μl containing 50 mM KCl, 10 mM Tris–HCl (pH 8.3), 1.5 mM MgCl_2_, 250 μM of each dNTP, 0.365 U of AB Taq polymerase (AB Analitica, Padova, Italy), 10 pmol of each primer and 50 ng of gDNA. A standard touchdown PCR protocol was used. The primer sequences for the SNaPShot™ reaction are available on demand. Capillary electrophoresis was performed on an ABI^®^ 3130 Genetic Analyzer (Thermo Fisher Scientific Inc.) and data were collected using GeneMapper v4.0 software (Thermo Fisher Scientific Inc.).

### *In silico* analysis

The putative functional relevance of the two analyzed missense variations was evaluated with PolyPhen-2 (http://genetics.bwh.harvard.edu/pph2/), SNAP (https://rostlab.org/services/snap/) and pMUT (http://mmb.pcb.ub.es/PMut/) software.

The putative effect of the other nucleotide variations on splice sites was evaluated using the ESEfinder scoring matrix (http://rulai.cshl.edu/cgi-bin/tools/ESE3/esefinder.cgi?process=home) and Human Splicing Finder (http://www.umd.be/HSF/).

### RNA isolation and quantitative real time (qRT)-PCR in GBM cell lines

Total RNA was purified from 18 GBM cell lines with the RNeasy Mini kit (Qiagen). One microgram of RNA was reverse-transcribed using random primers with the High-Capacity cDNA Reverse Transcription Kit (Thermo Fisher Scientific Inc.), according to the manufacturer's instructions.

qRT-PCR for *SEL1L*, *SOX2*, *NOTCH1* and *Gadd45β* genes was performed as already described [21]. Reactions were carried out using the Thermo Scientific Maxima SYBR Green/ROX qPCR Master Mix (Thermo Fisher Scientific Inc.) on a Rotor-Gene Q MDx instrument (Qiagen). The qRT-PCR conditions were the following: an initial denaturation step at 95°C for 10 min followed by 40 cycles at 95°C for 15 sec, 60°C for 30 sec, 72°C for 30 sec and final melting. Relative fold changes in the expression of genes of interest compared to the housekeeping gene *GADPH* were determined by the comparative ΔΔCt method. All assays were performed in triplicate.

*SEL1L* putative alternative transcripts were investigated in the same panel of 18 GBM cell lines by RT-PCR using specific primers: *SEL1L*-F: 5′-gaaggcagccaggatgaatcc-3′ and *SEL1L*-R: 5′-gcccccaagagctccaaatg-3′. The PCR conditions consisted of an initial denaturation step at 95°C for 3 min, followed by 32 cycles at 94°C for 1 min, 60°C for 1 min, 72°C for 1 min, with a final extension at 72°C for 5 min. All PCR products were electrophoresed on a 1.2% agarose gel and stained with ethidium bromide.

### Protein extraction and Western blotting analysis

Whole protein extracts from 11 cell lines (NO3 NS, NO4 NS, CTO3 NS, CV4 AC, CV6 AC, CV9 AC, CV10 AC, CV17 AC, NO3 AC, NO4 AC and CTO3 AC) were isolated in a lysis buffer containing 50 mM Tris HCl pH 7.6, 150 mM NaCl, 1% Nonidet P-40 and supplemented with protease inhibitors (Pierce Biotechnology, Rockford, IL, USA). After protein quantification by Bradford assay (Thermo Scientific Inc.), equal amounts of protein extracts were resolved by a 10% SDS-PAGE and transferred onto a PVDF membrane. Blots were probed with a mouse monoclonal [16] and a goat polyclonal (Santa Cruz Biotechnology Inc., Dallas, TX, USA) anti-SEL1L antibody and then treated with the appropriate horseradish peroxidase (HRP)-conjugated secondary antibody (GE Healthcare Life Science, MA, USA). Proteins were detected by enhanced chemiluminescence (Genespin, Milan, Italy). An anti-β-tubulin antibody (Sigma Aldrich Co., St. Louis, MO, USA) was used to normalize sample loading and transfer. Hybridizations were performed in a X-BlotP100 hybridization chamber (www.isenet.it).

### Drug treatments and cytotoxicity assay

VPA (from Sigma Aldrich Co.) was dissolved in sterile water, whereas TMZ, doxorubicin (DOX) and paclitaxel (PTX) (all from Sigma) were dissolved in 100% dimethylsulfoxide (DMSO) for stock solutions. Dilutions for all drug treatments were made extemporaneously in culture medium, so that the final concentration of DMSO never exceeded 0.3% (v/v).

The inhibitory effect of VPA was evaluated on six NS (NO3 NS, NO4 NS, CTO3 NS, CTO5 NS, CTO12 NS, CTO15 NS) and on three AC (NO3 AC, CTO3 AC and CTO15 AC). On the same cell lines we investigated the action of TMZ, DOX and PTX alone. The *in vitro* cytotoxicity of the four drugs, alone or combined, was evaluated assessing after exposure the number of viable cells by the 3-(4, 5-dimethylthiazol-2-yl)-2, 5-diphenyl-tetrazolium bromide (MTT) assay kit (Roche Diagnostics Corporation, Indianapolis, IL, USA) measuring formazan release at 570 nm by a microplate reader (Synergy HT, BioTek Instruments Inc., Winooski, VT, USA). For NS, cell counts were confirmed by Trypan Blue assay using a TC20 automated cell counter (Bio-Rad Laboratories, Hercules, CA, USA). Cytotoxicity was expressed as number of viable cells as percentage of control untreated cells. For each drug the IC50 value, that is the concentration required for 50% cell growth inhibition compared with untreated controls, was calculated by non-linear regression at 96 hours (h); based on these data, a concentration of 2 mM for VPA, 50 μM for TMZ, 1 μM for DOX and 50 nM for PTX were chosen for the following combination experiments.

To evaluate the effect of the association of VPA with the other three chemotherapeutics, cells were treated combining VPA with TMZ, DOX or PTX at the doses above mentioned and, after 96 h incubation, the cell viability was assessed as before described.

### Statistical methods

Association analysis was evaluated using 2 × 2 contingency tables by the Chi-square (χ^2^) or the two-tailed Fisher's exact test, as appropriate. The Student's *t*-test was used to compare the SNP rs12435998 genotype with the age at diagnosis.

OS was defined as the time between the histological diagnosis and patient's death or last follow-up. Patient alive at last follow-up were considered censored events. Survival curves were estimated using the Kaplan-Meier method and differences between them were compared by the Log-rank test (Mantel-Cox). Analysis was carried out by SPSS v21.0 software (SPSS Inc., Chicago, IL, USA).

For the cytotoxicity assays, the level of significance was determined by the Student's *t* test. *P* values < 0.05 were considered as statistically significant.

## SUPPLEMENTARY TABLE


