# Correction: *SEL1L* SNP rs12435998, a predictor of glioblastoma survival and response to radio-chemotherapy

**DOI:** 10.18632/oncotarget.26066

**Published:** 2018-08-24

**Authors:** Marta Mellai, Monica Cattaneo, Alessandra Maria Storaci, Laura Annovazzi, Paola Cassoni, Antonio Melcarne, Pasquale De Blasio, Davide Schiffer, Ida Biunno

**Affiliations:** ^1^ Neuro-Bio-Oncology Center/Policlinico di Monza Foundation, Vercelli 13100, Italy; ^2^ Institute for Genetic and Biomedical Research, National Research Council, Milan 20138, Italy; ^3^ Department of Medical Sciences, University of Turin/Città della Salute e della Scienza, Turin 10126, Italy; ^4^ Department of Neurosurgery, CTO Hospital/Città della Salute e della Scienza, Turin 10126, Italy; ^5^ IRCCS-Multimedica, Milan 20138, Italy; ^6^ ISENET Stem Cell Bank, Milan 20138, Italy

**This article has been corrected:** In this para, the term “somatic mutation” should be replaced with “genetic mutation” is given below:

The *SEL1L* gene spans more than 62.24 kb pairs within a “gene desert region” and displays only weak linkage disequilibrium pattern according to the HapMap CEU population data. A total of five single nucleotide genetic variants in the *SEL1L* gene (GenBank Reference sequence NM_005065) were selected (Table 6). Two of them (c.–366T > C and c.–354T > C) were first identified in the minimal promoter region of the *SEL1L* gene in lung carcinoma patients [47]. The c.341–88T > C genetic variant is a common SNP (rs12435998) within intron 3, containing potential binding sites for transcription factors involved in ER-induced stress, and it is a predicted splice site [23]. The c.485A > G (p.Asp162Gly) variant corresponds to the SNP rs11499034, maps in exon 4 encoding for the fibronectin type II domain (FN2), and affects a highly conserved amino acid residue [24]. The amino acid change from Asp to Gly may have a disruptive role in the collagen binding. The c.1972T > C (p.Ser658Pro) variant in exon 19 has been described as genetic mutation responsible for the progressive early-onset cerebellar ataxia in canine species [49]. Of note, the *SEL1L* gene is highly conserved between dog and human, with 98% identity at protein level (HomoloGene). The Ser658 residue located in the eleventh *SEL1L*-like repeat is completely conserved in all aligned vertebrates and maps within a functionally relevant domain for tumor growth inhibition [50].

Original article: Oncotarget. 2015; 6:12452-12467. https://doi.org/10.18632/oncotarget.3611

