# Supplementary material for: SEL1L SNP rs12435998, a predictor of glioblastoma survival and response to radio-chemotherapy
Source: Oncotarget. 2015 Apr 10;6(14):12452–67. doi: 10.18632/oncotarget.3611 (PMC4494950; doi:10.18632/oncotarget.3611)
Supplement: Supplementary file 1 [file oncotarget-06-12452-s001.pdf]

SUPPLEMENTARY TABLE

Supplementary Table S1. *SEL1L* nucleotide genetic variant genotypes in cell lines with respect to *MGMT* promoter hypermethylation and *TP53* mutation status

Cell line	SEL1L c. –366T > C	SEL1L c.–354T > C	SEL1L c.341–88T > C (rs12435998)	SEL1L c.485A > G (rs11499034)	SEL1L c.1792T > C (p.Ser658Pro)	MGMT methylation	TP53 mutation	COSMIC ID	SNP ID
**CV1 NS**	TT	TT	TC	AA	TT	UnMeth	Wild type / p.Pro72Arg		rs1042522
**CV2 AC**	TT	TT	TT	AA	TT	UnMeth	Wild type / p.Pro72Arg		rs1042522
**CV3 AC**	TT	TT	TT	AA	TT	UnMeth	p.Arg213Arg / IVS7–35A > G	COSM249885	rs1800372
**CV4 AC**	TT	TT	TC	AA	TT	UnMeth	Wild type		
**CV6 AC**	TT	TT	TC	AA	TT	UnMeth	Wild type		
**CV7 NS**	TT	TT	TT	AA	TT	Meth	p.Cys176Tyr* / p.Pro72Arg	COSM10687	rs1042522
**CV8 AC**	TT	TT	TT	AA	TT	UnMeth	Wild type / p.Pro72Arg		rs1042522
**CV9 AC**	TT	TT	TC	AA	TT	UnMeth	p.Arg213Arg / p.Pro72Arg	COSM249885	rs1800372, rs1042522
**CV10 NS**	TT	TT	TC	AA	TT	Meth	Wild type		
**CV10 AC**	TT	TT	TC	AA	TT	Meth	Wild type		
**CV13 NS**	TT	TT	TT	AA	TT	UnMeth	Wild type, IVS4.+5A/G*		
**CV17 NS**	TT	TT	TT	AA	TT	Meth	Wild type		
**CV17 AC**	TT	TT	TT	AA	TT	UnMeth	Wild type		
**CV20 NS**	TT	TT	TC	AG	TT	Meth	p.Arg158Lys* / p.Arg273Cys* / p.Pro72Arg	COSM10714	rs1042522
**CV21 NS**	TT	TT	TT	AA	TT	Meth	p.Arg213Arg	COSM249885	rs1800372
**CV21 AC**	TT	TT	TT	AA	TT	UnMeth	p.Arg213Arg	COSM249885	rs1800372
**NO2 AC**	TT	TT	TC	AA	TT	UnMeth	Wild type		
**NO3 NS**	TT	TT	TT	AA	TT	UnMeth	Wild type		

(*Continued*)

Cell line	SEL1L c. –366T > C	SEL1L c.–354T > C	SEL1L c.341–88T > C (rs12435998)	SEL1L c.485A > G (rs11499034)	SEL1L c.1792T > C (p.Ser658Pro)	MGMT methylation	TP53 mutation	COSMIC ID	SNP ID
**NO3 AC**	TT	TT	TT	AA	TT	UnMeth	Wild type / p.Pro72Arg		rs1042522
**NO4 NS**	TT	TT	TC	AA	TT	Meth	p.His179Tyr / p.Pro72Arg	COSM10768	rs1042522
**NO4 AC**	TT	TT	TC	AA	TT	UnMeth	Wild type		
**NO6 NS**	TT	TT	TT	AA	TT	UnMeth	Wild type		
**CTO3 NS**	TT	TT	CC	AA	TT	Meth	p.Cys275Tyr*	COSM10893	
**CTO3 AC**	TT	TT	CC	AA	TT	Meth	p.Cys275Tyr*	COSM10893	
**CTO5 NS**	TC	TC	TT	AA	TT	UnMeth	Wild type / p.Pro72Arg		rs1042522
**CTO5 AC**	TC	TC	TT	AA	TT	UnMeth	Wild type / p.Pro72Arg		rs1042522
**CTO12 NS**	TT	TT	TT	AA	TT	Meth	c.376–5C > A / p.Pro72Arg		rs1042522
**CTO12 AC**	TT	TT	TT	AA	TT	nd	nd		
**CTO15 NS**	TT	TT	TC	AA	TT	UnMeth	p.Tyr220Cys*	COSM10758	
**CTO15 AC**	TT	TT	TC	AA	TT	UnMeth	nd		
**U87-MG NS**	TT	TT	TT	AA	TT	Meth	Wild type		
**U87-MG AC**	TT	TT	TT	AA	TT	Meth	Wild type		
**010627 NS**	TT	TT	TC	AA	TT	Meth	p.L130I / p.P278S**	Not found, COSM10939	
**010627 AC**	TT	TT	TC	AA	TT	Meth	p.L130I / p.P278S**	Not found, COSM10939	

(*Continued*)

Cell line	SEL1L c. –366T > C	SEL1L c.–354T > C	SEL1L c.341–88T > C (rs12435998)	SEL1L c.485A > G (rs11499034)	SEL1L c.1792T > C (p.Ser658Pro)	MGMT methylation	TP53 mutation	COSMIC ID	SNP ID
**GBM2**	TT	TT	TT	AA	TT	nd	nd		
**G144**	TT	TT	TT	AA	TT	nd	nd		
**G166**	TT	TT	TT	AA	TT	nd	nd		
**G179**	TT	TT	TC	AA	TT	nd	nd		
**GliNS2**	TT	TT	TT	AA	TT	nd	nd		
**CB660**	TT	TT	TT	AA	TT	nd	nd		
**H9**	TT	TT	TT	AA	TT	nd	nd		

*Note: Somatic origin verified
**Constitutional DNA not available.

Abbreviations: SEL1L, suppressor of Lin-12-like; GBM, glioblastoma multiforme; MGMT, O^6-methylaguanine methyl-transferase; TP53, tumor protein p53; NS, neurospheres; AC, adherent cells; Meth, methylated; UnMeth, unmethylated; nd, not determined; COSMIC, Catalogue of Somatic Mutations in Cancers; SNP, single nucleotide polymorphism.
